# Symptom Overlap and Screening for Symptoms of Attention-Deficit/Hyperactivity Disorder and Psychosis Risk in Help-Seeking Psychiatric Patients

**DOI:** 10.3389/fpsyt.2017.00206

**Published:** 2017-10-30

**Authors:** Salvatore Corbisiero, Anita Riecher-Rössler, Jacqueline Buchli-Kammermann, Rolf-Dieter Stieglitz

**Affiliations:** ^1^Clinical Psychology and Psychiatry, University of Basel Psychiatric Hospital, Basel, Switzerland; ^2^Center for Gender Research and Early Detection, University of Basel Psychiatric Hospital, Basel, Switzerland; ^3^Department of Psychology, Division of Clinical Psychology and Psychiatry, University of Basel, Basel, Switzerland

**Keywords:** attention-deficit/hyperactivity disorder, psychosis risk, prodrome, schizophrenia, screening

## Abstract

Symptoms of attention-deficit/hyperactivity disorder (ADHD) and psychosis risk share features which might represent an early vulnerability marker for schizophrenia. Early detection of individuals with this symptomatic overlap is relevant and may assist clinicians in their decision making for diagnosis and treatment. This study sought to analyze the capability of different instruments in the screening of patients for ADHD symptoms or at psychosis risk, assess their classification accuracy, and describe the extent of symptoms overlap between them. 243 adult patients completed one instrument screening for ADHD and two instruments screening for psychosis risk symptoms [*Adult ADHD Self-Report Scale Symptom Checklist* (ASRS-v1.1); *Prodromal Questionnaire Brief Version* (PQ-16); *Self-Screen Prodrome* (SPro)]. The ability of these instruments to distinguish between the symptomaticity of these patients appears modest. The most satisfactory scale to identify subjects at psychosis risk was SPro with its subscale *psychosis risk*. ASRS-v1.1 showed good reliability in assessing individuals as not having ADHD symptoms and had higher probability to achieve its own and the cut-off of another questionnaire. Subjects having symptoms of psychosis risk and ADHD showed elevated symptomatology. Reliable instruments capable of separating ADHD symptoms from those of psychosis risk are needed to better identify the symptomatic overlap of this two conditions.

## Introduction

Attention-deficit/hyperactivity disorder (ADHD) and schizophrenia are two neurodevelopmental disorders associated with a certain symptomatic overlap (1). Common features of both conditions are attentional difficulties, inner tension, emotional dysregulation, and disorganized behavior (2–6). While these problems are part of the symptoms of ADHD, they are more likely to arise at the level of associated features in schizophrenia (6). Neurocognitive and developmental deficits commonly precede schizophrenia (7), making it not only difficult to distinguish between the psychopathologies of the two disorders but also to distinguish psychosis risk from ADHD symptoms. Studies on the clinical overlap of these conditions or on the co-occurrence of ADHD and psychosis risk symptoms are relatively rare and inconsistent (5–7).

ADHD and schizophrenia are often accompanied by impairment in major life activities (8–11). Therefore, early identification of individuals with ADHD and those at risk of psychosis is imperative. The majority of individuals with a high risk of psychosis show ADHD symptoms (3, 5), which might be an early vulnerability marker for schizophrenia (12), especially also because individuals developing a schizophrenia spectrum disorder or children at genetic risk of schizophrenia have been more often diagnosed with an ADHD in childhood (12–14). Furthermore, a diagnosis of ADHD in childhood, compared to unipolar depression, was found to be most predictive of schizophrenia in adulthood (15). In their long-term prospective follow-up study, Dalsgaard et al. (16) showed that children and adolescents with a diagnosed ADHD were at a 4.3 times higher risk of developing schizophrenia in adulthood compared to controls.

In particular, executive function deficits (i.e., sustained attention, working memory, and vigilance) seem to reflect symptoms shared between psychosis risk and ADHD (3, 17), although findings of existing studies are inconsistent and difficult to compare. Attention deficits were frequently identified in subjects at psychosis risk symptoms (12, 17). However, it has also been reported that there are no significant differences in measures of cognitive function between individuals at psychosis risk and ADHD (18).

Hyperactivity and impulsivity, core symptoms of ADHD (19, 20), are also observed in individuals at psychosis risk (7, 12, 21). Similar to ADHD patients (22), patients at psychosis risk show a progressively lower level of frustration tolerance and higher emotional lability (4, 17). In addition, individuals at psychosis risk have limited emotional regulation skills (4) similar to those of ADHD patients (22).

All these findings, although sparse and inconsistent, suggest that symptoms of ADHD, and psychosis risk share some common features. There is a need to better understand this overlap which may help clinicians in their decision-making for diagnosis and treatment.

Effective screening for ADHD and for symptoms of psychosis risk could reduce inadequate treatment prolonging the suffering of patients. Generally, the screening for a disorder is the first step of the diagnostic process (23, 24) with the aim of identifying individuals at high risk of a particular disease and patients already suffering from the disorder (25). An adequate screening instrument should meet application-related, content, and methodical criteria. The screening process should be quick and easy to perform and allow an interpretation of the test results by means of cut-off values (COV) to separate healthy from clinical individuals. Screening tools satisfying these objectives are self-rating instruments addressing all relevant criteria of a specific disorder, and their efficacy and utility defined by several psychometric parameters: *reliability, validity, sensitivity*, and *specificity*.

*Sensitivity* (or true positive rate; SENS) refers to the probability of a positive test result being correctly identified in a person with a specific disorder. *Specificity* (or true negative rate; SPEC) measures the probability of a negative test result being correctly identified in a healthy person not suffering from a specific disorder (25). Two further parameters are relevant in this context: *positive predictive value* (PPV) and *negative predictive value* (NPV). The first measures the level of probability at which individuals with a positive test value actually do suffer from a defined condition. The second measures the level of probability at which individuals with a negative test value do not actually suffer from a defined condition (25). A positive screening value does not, however, obviate the need for further diagnostic procedures but calls for a specific diagnostic procedure able to confirm or dismiss the suspicion of a particular disorder.

To further understand the potential overlap of ADHD and psychosis risk symptoms, the aim of this study was to analyze the capability of three different screening instruments in identifying patients at psychosis risk or with ADHD symptomatology and specify the classification accuracy of these instruments. A second analysis focused on the similarity of criteria present in these instruments, i.e., two screenings for psychosis risk symptoms [*Prodromal Questionnaire Brief Version* (PQ-16) (26); *Self-Screen Prodrome* (Spro) (27)] and one screening for ADHD symptoms [*Adult ADHD Self-Report Scale Symptom Checklist* (ASRS-v1.1) (28, 29)]. A further objective was to estimate odds ratios (OR) for each instrument. We analyzed the OR to achieve the COV of each different scale and tested how strongly the presence or absence of psychosis risk and ADHD symptoms is associated with the presence or absence of ADHD and psychosis risk symptoms in a clinical population. We hypothesized:

(1) that the overlap of the measured scales between the two psychosis risk screenings is greater than that between the ADHD instrument and the two psychosis screenings.
(2) that a stronger association exists between the two instruments screening for psychosis risk symptoms than between those two instruments and the one screening for ADHD.
(3) that psychosis risk symptoms are not to distinguish from ADHD symptoms with reliable and valid instruments screening symptoms for psychosis risk or ADHD.

## Materials and Methods

### Participants

The sample included a total of 243 patients recruited across eight (mainly outpatient) clinics that belonging to the University of Basel Psychiatric Clinics (UPC) in Basel (Switzerland) between March 2013 and August 2014. Participants gave written informed consent. The study was formally approved by the Ethics Committee of Basel (Ethikkommission Nordwest- und Zentralschweiz). There were 155 (63.8%) males and 88 (36.2%) females with a gender difference [χ^2^ (1, *N* = 243) = 18.47, *p* < 0.001]. Participating individuals were between 18 and 75 years old (*M* = 35.83, *SD* = 11.38) and being treated in one of the eight clinics at UPC with group differences in gender [χ^2^(7, *N* = 243) = 23.46, *p* = 0.001] and age [ANOVA: *F*(7, 243) = 8.86, *p* < 0.001]. The distribution of the sample as to gender and age across the different clinics is presented in Table 1.

**Table 1:** Distribution of the sample (*N* = 243) in gender and age in the different clinics.

	Clinics at University of Basel Psychiatric Clinics (UPC)							
	ZA^a^	ZDK^b^	VTA^c^	FAM^d^	ADS^e^	U3^f^	ZASS^g^	ZFM^h^
*n* (%)	42 (17.3)	22 (9.1)	59 (24.3)	5 (2.1)	84 (34.6)	8 (3.3)	13 (5.3)	10 (4.1)
Genders								
Female: *n* (%)	11 (12.5)	8 (9.1)	35 (39.8)	0 (0)	25 (28.4)	1 (1.1)	6 (6.8)	2 (2.3)
Male: *n* (%)	31 (20.0)	14 (9.0)	24 (15.5)	5 (3.2)	59 (38.1)	7 (4.5)	7 (4.5)	8 (5.2)
Age: M (SD)	34.36 (1.59)	26.59 (2.19)	31.76 (1.34)	34.00 (4.60)	42.20 (1.12)	38.63 (3.63)	34.92 (2.85)	32.70 (3.25)

*^a^Central Patient Admission [Zentrale Aufnahme]*.

*^b^Center for Diagnostic and Crisis Intervention [Zentrum für Diagnostik und Krisenintervention]*.

*^c^Ambulance Behavioral Therapy [Verhaltenstherapie Ambulanz]*.

*^d^Forensic Ambulance [Forensische Ambulanz]*.

*^e^Ambulance Addiction Services [Ambulanz für Sucht]*.

*^f^Inpatient Unit for Substance Dependence and Addiction [Stationäre Abteilung für Abhängigkeit und Sucht]*.

*^g^Center for Affective, Stress and Sleep Disorder [Zentrum für Affektive-, Stress- und Schlafstörungen]*.

*^h^Center for Psychotic Disorders and Transcultural Psychiatry [Zentrum für psychotische Erkrankungen und transkulturelle Psychiatrie]*.

### Procedure

Three different self-rating scales assessing ADHD or psychotic symptoms were distributed mainly to outpatient clinics (seven outpatient, one inpatient) at UPC. The medical staff, primarily psychiatrists and psychologists, were briefed about the study and passed the questionnaires to participants during the process of admission in the different clinics. Details about the study, informed consent forms to be signed and instructions on how to complete the scales were given to participants in written form. The completed questionnaires were collected regularly, evaluated, and returned to the attending psychiatrists and psychologists. For patients who exceeded the COV, referral to our specialized ADHD or early detection of psychosis clinic was recommended.

### Instruments

#### Adult ADHD Self-Report Scale Symptom Checklist [ASRS-v1.1 (28, 29)]

The World Health Organization self-rating scale is based on the 18 DSM-IV ADHD criteria and may provide information suggesting the need of a more in-depth diagnostic evaluation. Six (part A) of the 18 questions were found the most predictive of symptoms consistent with ADHD in adulthood (29). The 12 remaining questions (part B) provide additional cues on the patient’s symptoms. The instrument with its five-point scale from never (0) to very often (4) takes about 5 min to complete. The total score is calculated from the sum value of each item. The higher the score, the more pronounced the symptoms are. In addition to the sum score of the checklist, the scores for the two subscales *inattention* and *hyperactivity/impulsivity* can be calculated. Part A helps to identify patients’ symptoms correctly. Part B records the severity of the symptoms identified. Part A is seen as the short version of the checklist [ASRS-6 (29)], consisting of four items from the *inattention* and two from the *hyperactivity/impulsivity* subscale. By exceeding a cut-off value of 4 in ASRS-6, an adult ADHD is likely. Kessler et al. (29) established good psychometric properties for the screener (ASRS-6) and the full version (ASRS-18): sensitivity: 68.7 versus 56.3%, specificity: 99.5 versus 98.3%, total classification accuracy: 97.9 versus 96.2%, and Cohen’s κ: 0.76 versus 0.58. Buchli-Kammermann et al. (30) determined the psychometric quality of the German version, obtaining similar values for both versions (ASRS-6 versus ASRS-18): sensitivity: 66.6 versus 72.3%; specificity: 64.9 versus 68.1%, Cronbach’s α: 0.73 versus 0.89. For the screening process the ASRS-6 proved particularly relevant. Ramos-Quiroga et al. (31) revealed a new strategy in scoring symptoms by using a quantitative ranking between 0 and 24 points for either subscale (*inattention* and *hyperactivity/impulsivity*) with a cut-off value of 12 points (sensitivity: 96.7%, specificity: 91.1%, Cohen’s κ: 0.88). For the current study, the short (ASRS-6) and full version (ASRS-18) of this screening instrument were used. The cut-off value of 12 points in the full version (ASRS-18) proposed by Ramos-Quiroga et al. (31) was adopted.

#### Prodromal Questionnaire Brief Version [PQ-16 (26)]

The self-rating scale is a brief version of the *Prodromal Questionnaire* with 92 items (32) able to identify subjects at psychosis risk on a two-point scale (true/false). PQ-16 consists of three subscales: *positive* (perceptual abnormalities/hallucinations; 9 items), *disorganized* (unusual thought content/delusional ideas/paranoia; 5 items), and *negative symptoms* (excessive social anxiety/avolition; 2 items). A COV of ≥6 has high sensitivity (87%) and high specificity (87%) with a PPV of 44%, and an internal consistency of Cronbach’s α 0.774.

#### Self-Screen Prodrome [Spro (27)]

This self-rating scale differentiates between healthy individuals, individuals with psychosis or at psychosis risk, and patients with other mental disorders. It has 32 items on a two-point scale (true/false): items 1–24 measure *psychopathological changes* with nonspecific, negative and positive symptoms of a psychosis; items 25–29 determine the level of *psychosocial functioning*; items 30–32 detect *psychosis risk deriving from a genetic vulnerability or consumption of substances*. Six items frame the subscale *psychosis risk* and are seen as decisive prodromal symptoms. If at least two items are answered positively, an in-depth diagnostic procedure in a special consultation for the early detection of psychoses is recommended.

An SPro total score of ≥6 indicates the presence of a mental disorder with a sensitivity of 85% and a specificity of 91%. A psychosis risk subscale score ≥2 distinguishes between psychosis-(risk)-individuals and outpatients with another mental disorder with a sensitivity of 85% and a specificity of 39%. The internal consistency of the scale for healthy individuals (Cronbach’s α = 0.75), psychosis-(risk)-individuals (Cronbach’s α = 0.89), and individuals with another mental disease (Cronbach’s α = 0.89) is acceptable. In particular, the sum score and the subscale *psychosis risk* of this instrument were taken into consideration.

### Data Analysis

Data analyses were conducted using SPSS Version 23. First, the sample was described regarding probable differences in gender, age, clinics, and the four diagnostic category groups: (01) no indication of symptoms; (02) indication of ADHD symptoms; (03) indication of psychosis symptoms; and (04) indications of ADHD and psychosis symptoms. To analyze a potential symptoms overlap several χ^2^-tests identified the number of cases (individuals under or above the COV) detected by the three instruments used. The internal consistency for all scales was estimated with Cronbach’s α, the relationship between them with Spearman’s correlation coefficients. Analyses of the Receiver Operating Characteristics (ROC) were performed to examine SENS, SPEC, PPV, NPV, and the area under the curve (AUC). The ROC curve analysis is used to compare the diagnostic performance of the different screening instruments. The AUC measures the discrimination, i.e., the ability of an instrument to correctly classify individuals with and without ADHD or psychotic risk symptoms. The OR to achieve not only the COV of one instrument but also that of another were analyzed with χ^2^-tests. The characteristics of the symptoms overlap of the different instruments in the four diagnostic category groups were visualized with exploratory data analysis.

## Results

### Descriptive Statistics

The analyses of the questionnaires showed that 35.8% (*n* = 87) of the sample had no symptoms of psychosis risk or ADHD, 3.3% (*n* = 8) presented with ADHD and 40.7% (*n* = 99) with psychosis risk symptoms only; 20.2% (*n* = 49) displayed symptoms of ADHD as well as psychosis risk symptoms. The comparisons between the four established diagnostic categories were statistically not significant in gender [χ^2^(3, *N* = 243) = 2.44, *p* = 0.49], age [ANOVA: *F*(3, 243) = 0.89, *p* = 0.447], and clinics [χ^2^(21, *N* = 243) = 27.34, *p* = 0.16].

### Differences in Frequency of Cases below or above the COV of ASRS-v1.1, SPro, and PQ-16

To better understand the relationship between the instruments (subscales and sum scales) used, different χ^2^-tests were conducted (Table 2). All comparisons were statistically significant except that between ASRS-18 and SPro. The observed frequencies of individuals achieving or exceeding the COV were nearly all statistically significantly higher than the expected frequencies, as were the observed frequencies of individuals not achieving or exceeding the COV. The short version of the ASRS-v1.1 (ASRS-6) shared one of the highest frequencies with the other subscales and sum scales. The observed frequencies of individuals achieving or exceeding the COV in ASRS-6 and in the SPro subscale *psychosis risk* (*n* = 71, 30.3%) and SPro (*n* = 69, 29.5%) were most prevalent.

**Table 2:** Number of cases below or above the cut-off value (COV) of the different scales.

Scale			ASRS-18		SPro *psychosis risk*		SPro		PQ-16^e^	
			COV^f^ < 12	COV ≥ 12	COV < 2	COV ≥ 2	COV < 6	COV ≥ 6	COV < 6	COV ≥ 6
ASRS-6^a^	COV < 4	Count	155	4	62	93	37	118	130	28
		Expected count	132.7	26.3	46.4	108.6	31.1	123.9	117.8	40.2
		% of Total	64.0	1.7	26.5	39.7	15.8	50.4	54.2	11.7
	COV ≥ 4	Count	47	36	8	71	10	69	49	33
		Expected count	69.3	13.7	23.6	55.4	15.9	63.1	61.2	20.8
		% of Total	19.4	14.9	3.4	30.3	4.3	29.5	20.4	13.8

ASRS-18^b^	COV < 12	Count			67	129	43	153	161	40
		Expected count			58.6	137.4	39.4	156.6	149.9	51.1
		% of Total			28.2	55.1	18.4	65.4	67.1	16.7
	COV ≥ 12	Count			3	35	4	34	18	21
		Expected count			11.4	26.6	7.6	30.4	29.1	9.9
		% of Total			1.3	15.0	1.7	14.5	7.5	8.8

SPro^c^ *psychosis risk*	COV < 2	Count					37	33	64	5
		Expected count					14.0	56.0	51.5	17.5
		% of Total					15.7	14.0	27.6	2.2
	COV ≥ 2	Count					10	155	109	54
		Expected count					33.0	132.0	121.5	41.5
		% of Total					4.3	66.0	47.0	23.3

SPro^d^	COV < 6	Count							44	2
		Expected count							34.3	11.7
		% of Total							19.0	0.9
	COV ≥ 6	Count							129	57
		Expected count							138.7	47.3
		% of Total							55.6	24.6

*^a^Adult attention-deficit/hyperactivity disorder (ADHD) Self-Report Scale Symptom Checklist: part A (6 items)*.

*^b^Adult ADHD Self-Report Scale Symptom Checklist: parts A & B (18 items)*.

*^c^Self-Screen Prodrome: subscale psychosis risk*.

*^d^Self-Screen Prodrome*.

*^e^Prodromal Questionnaire Brief Version*.

*^f^Cut-off value*.

*χ^2^_ASRS-6 – ASRS-18_ (1, N = 242) = 65.982, p < 0.001*.

*χ^2^_ASRS-6 – SPro psychosis risk_ (1, N = 234) = 22.274, p < 0.001*.

*χ^2^_ASRS-6 – SPro_ (1, N = 234) = 4.099, p < 0.05*.

*χ^2^_ASRS-6 – PQ-16_ (1, N = 240) = 14.445, p < 0.001*.

*χ^2^_ASRS-18 – SPro psychosis risk_ (1, N = 234) = 10.492, p = 0.001*.

*χ^2^_ASRS-18 – SPro_ (1, N = 234) = 2.583, p = 0.108*.

*χ^2^_ASRS-18 – PQ-16_ (1, N = 240) = 19.854, p < 0.001*.

*χ^2^_SPro psychosis risk – SPro_ (1, N = 235) = 67.270, p < 0.001*.

*χ^2^_SPro psychosis risk – PQ-16_ (1, N = 232) = 17.125, p < 0.001*.

*χ^2^_SPro – PQ-16_ (1, N = 232) = 13.449, p < 0.001*.

### Internal Consistency, Convergent, and Divergent Validity of ASRS-v1.1, SPro, and PQ-16

The internal consistency was estimated for all scales. SPro showed the highest with a Cronbach’s α of 0.959, succeeded by ASRS-18 (α = 0.898), ASRS-18 *inattention* (α = 0.847), ASRS-18 *hyperactivity/impulsivity* (α = 0.841), PQ-16 (α = 0.826), SPro *psychosis risk* (α = 0.800), and ASRS-6 (α = 0.702).

The correlations of the total scores are presented in Table 3 with instruments correlating significantly (*p* < 0.01) among themselves. As expected, the correlations between scales measuring similar aspects {e.g., SPro versus SPro *psychosis risk* [*r_s_*(234) = 0.826] or PQ-16 [*r_s_*(232) = 0.600]} were strong. By contrast, ASRS-18 versus SPro *psychosis risk* or SPro, measuring different areas of symptomaticity, showed a small correlation only [*r_s_*(233) = 0.380 and *r_s_*(234) = 0.474].

**Table 3:** Spearman correlations of the total score of the different scales.

Scale	ASRS-6	ASRS-18	SPro *psychosis risk*	SPro	PQ-16^e^
ASRS-6^a^		0.835**	0.380**	0.474**	0.421**
ASRS-18^b^			0.499**	0.604**	0.526**
SPro^c^ *psychosis risk*				0.826**	0.536**
SPro^d^					0.600**

*^a^Adult attention-deficit/hyperactivity disorder (ADHD) Self-Report Scale Symptom Checklist: part A (6 items)*.

*^b^Adult ADHD Self-Report Scale Symptom Checklist: parts A & B (18 items)*.

*^c^Self-Screen Prodrome: subscale psychosis risk*.

*^d^Self-Screen Prodrome*.

*^e^Prodromal Questionnaire Brief Version*.

**p < 0.01.

### Receiver Operating Characteristics Analyses of ASRS-v1.1, SPro, and PQ-16

The sum score of every scale was compared with the COV of the subscales and sum scales of the instruments used. The results are shown in Table 4. The AUC was significant in the comparisons of all scales. The highest SENS was exemplified by the sum scale of SPro and its subscale *psychosis risk*, which was found to be the best scale to identify individuals at risk of symptoms of psychosis. The ASRS-v1.1 (ASRS-18) had the highest values of SPEC, indicating the capacity to detect correctly individuals not having symptoms of ADHD.

**Table 4:** Classification accuracy of the different scales, i.e., sensitivity (SENS), specificity (SPEC), positive predictive value (PPV), negative predictive value (NPV), area under the curve (AUC), and confidence interval (CI).

	SENS^a^	SPEC^b^	PPV^c^	NPV^d^	AUC^e^	CI^f^
**ASRS-6^g^**						
ASRS-18^h^	90.00	76.73	43.37	97.48	0.923***	0.886–0.959
SPro *psychosis risk*^i^	43.29	88.57	89.87	40.00	0.746***	0.679–0.813
SPro^j^	36.90	78.72	87.34	23.87	0.690***	0.599–0.782
PQ-16^k^	54.10	72.62	40.24	82.28	0.721***	0.649–0.793

**ASRS-18**						
ASRS-6	43.37	97.48	90.00	76.73	0.906***	0.870–0.943
SPro *psychosis risk*	21.34	95.71	92.11	34.18	0.793***	0.731–0.854
SPro	18.18	91.49	89.47	21.94	0.749***	0.665–0.833
PQ-16	34.43	89.94	53.85	80.10	0.749***	0.679–0.819

**SPro *psychosis risk***						
ASRS-6	89.87	40.00	43.29	88.57	0.671***	0.600–0.742
ASRS-18	92.11	34.18	21.34	95.71	0.694***	0.597–0.790
SPro	82.45	78.72	93.94	52.86	0.839***	0.777–0.902
PQ-16	91.53	36.99	33.13	92.75	0.746***	0.671–0.821

**SPro**						
ASRS-6	87.34	23.87	36.90	78.72	0.709***	0.641–0.778
ASRS-18	89.47	21.94	18.18	91.49	0.739***	0.644–0.834
SPro *psychosis risk*	93.94	52.86	82.45	78.72	0.851***	0.797–0.905
PQ-16	96.61	25.43	30.65	95.65	0.763***	0.693–0.832

**PQ-16**						
ASRS-6	40.24	82.28	54.10	72.62	0.683***	0.612–0.753
ASRS-18	53.85	80.10	34.43	89.94	0.773***	0.694–0.852
SPro *psychosis risk*	33.13	92.75	91.53	36.99	0.722***	0.654–0.790
SPro	30.65	95.65	96.61	25.43	0.736***	0.660–0.811

*^a^Sensitivity*.

*^b^Specificity*.

*^c^Positive predictive value*.

*^d^Negative predictive value*.

*^e^Area under the curve*.

*^f^Confidence interval*.

*^g^Adult attention-deficit/hyperactivity disorder (ADHD) Self-Report Scale Symptom Checklist: part A (6 items)*.

*^h^Adult ADHD Self-Report Scale Symptom Checklist: parts A & B (18 items)*.

*^i^Self-Screen Prodrome: subscale psychosis risk*.

*^j^Self-Screen Prodrome*.

*^k^Prodromal Questionnaire Brief Version*.

***p > 0.001.

### Odds Ratios: Achievement of Different COV of ASRS-v1.1, SPro, and PQ-16

The association between the COV of the different scales was represented with OR. The aim was to estimate the OR to achieve the COV of one scale in relation to the achievement of the COV of another scale. These results are introduced in Table 5. The ASRS-18, in comparison to its short form ASRS-6, had a higher probability of achieving the COV of another instrument: The probability for the COV of the ASRS-18 to achieve the COV of SPro subscale *psychosis risk* has an OR of 6.059, and an OR of 4.696 for the achievement of the COV of the PQ-16.

**Table 5:** Odds ratios (OR) to achieve the cut-off values (COV) of different scales.

Scale		ASRS-18	SPro *psychosis risk*	SPro	PQ-16^e^
ASRS-6^a^	OR^f^	29.681	5.917	2.164	3.127
	95% CI^g^				
	Lower	10.046	2.663	1.013	1.714
	Upper	87.692	13.148	4.622	5.705

ASRS-18^b^	OR		6.059	2.389	4.696
	95% CI			
	Lower		1.797	0.803	2.289
	Upper		20.432	7.104	9.633

SPro^c^ *psychosis risk*	OR			17.379	6.341
	95% CI				
	Lower			7.862	2.412
	Upper			38.415	16.674

SPro^d^	OR				9.9721
	95% CI			
	Lower				2.278
	Upper				41.480

*^a^Adult attention-deficit/hyperactivity disorder (ADHD) Self-Report Scale Symptom Checklist: part A (6 items)*.

*^b^Adult ADHD Self-Report Scale Symptom Checklist: parts A & B (18 items)*.

*^c^Self-Screen Prodrome: subscale psychosis risk*.

*^d^Self-Screen Prodrome*.

*^e^Prodromal Questionnaire Brief Version*.

*^f^Odds ratio*.

*^g^Confidence interval*.

### Exploratory Data Analysis: Symptoms Overlap of ASRS-v1.1, SPro, and PQ-16

**Figure 1:**
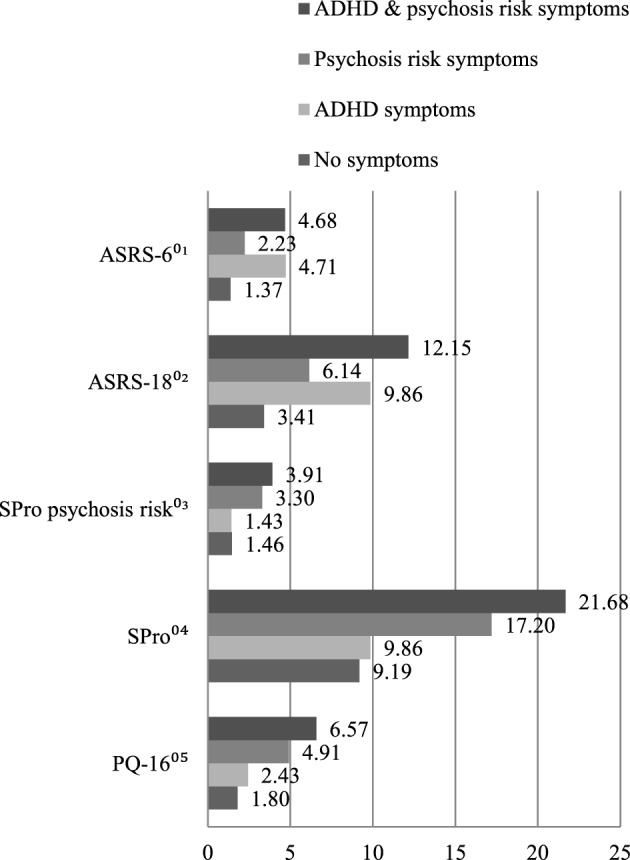
Symptomatic overlap between the four diagnostic groups: means of the total score of ^01^Adult attention-deficit/hyperactivity disorder (ADHD) Self-Report Scale Symptom Checklist: part A (6 items); ^02^Adult ADHD Self-Report Scale Symptom Checklist: parts A & B (18 items); ^03^Self-Screen Prodrome: subscale *psychosis risk*; ^04^Self-Screen Prodrome; ^05^Prodromal Questionnaire Brief Version.

**Figure 2:**
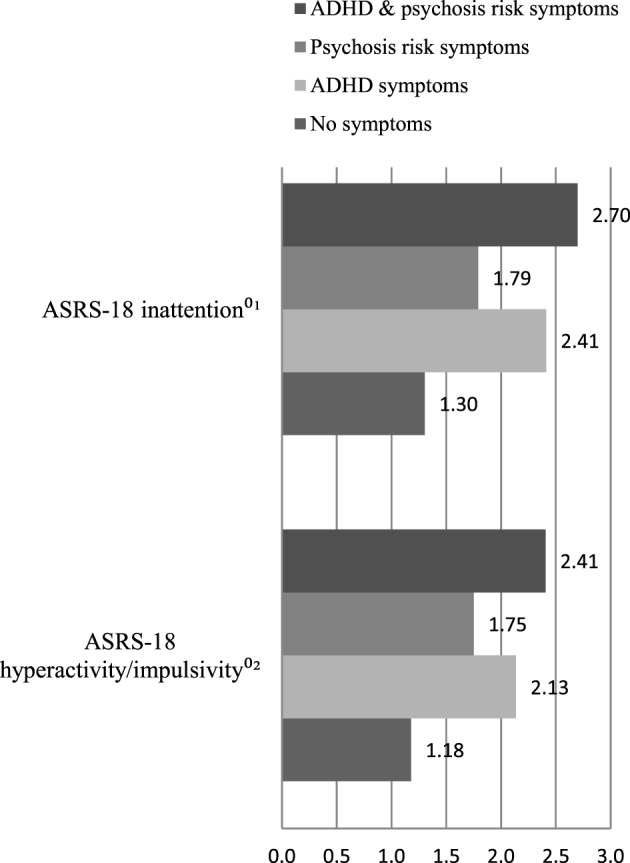
Symptomatic overlap between the four diagnostic groups: means of the total score of ^01^Adult attention-deficit/hyperactivity disorder (ADHD) Self-Report Scale Symptom Checklist: subscale inattention; ^02^Adult ADHD Self-Report Scale Symptom Checklist: subscale hyperactivity/impulsivity.

The means of the total score of the different measures were analyzed and compared in the four diagnostic groups: (01) no indication of symptoms; (02) indication of ADHD symptoms; (03) indication of psychosis symptoms; and (04) indications of ADHD and psychosis symptoms. Individuals with ADHD as well as psychosis symptoms had higher means in nearly every instrument. Only in the ASRS-6 did subjects with an indication of ADHD symptoms achieve a slightly higher mean (cf. Figure 1). The analyses of the ASRS-18 subscales of *inattention* and *hyperactivity/impulsivity*, presented in Figure 2, showed higher means for the diagnostic group (4) (subjects with ADHD and psychosis symptoms).

## Discussion

The current study analyzes the ability of three different instruments to screen patients at risk of psychosis or ADHD and the symptomatic overlap measured with these questionnaires. Overall, the ability of ASRS-v1.1, SPro, and PQ-16 to distinguish clearly between patients with these symptoms is found to be modest. The most satisfactory scale to identify subjects with a risk of psychosis was SPro and its subscale *psychosis risk*. ASRS-18 showed good ability to detect correctly individuals not presenting with symptoms of ADHD. However, this instrument demonstrated a higher probability to achieve, besides its own COV, the COV of another questionnaire. Individuals showing symptoms of psychosis as well as ADHD had elevated symptomatology in nearly all instruments. With the instruments analyzed here, it appears difficult in a clinical sample to differentiate clearly between ADHD and psychotic symptoms.

The biggest overlap between individuals achieving the COV of two different questionnaires was not found, as expected, between scales defining similar aspects but between the SPro subscale *psychosis risk* and ASRS-6, which proved to be even higher than that found between ASRS-6 and ASRS-18. Marwaha et al. (7) demonstrated a similar association between ADHD and psychosis with a higher score of ASRS-6 being significantly associated with psychosis. Overall, ASRS-6 shared the highest case frequencies with the other scales. The overlap between PQ-16 and both versions of ASRS-v1.1 was, however, as expected, the smallest. SPro and ASRS-v1.1 seem to share many more aspects of psychotic and ADHD symptoms than PQ-16 and ASRS-v1.1: while in SPro symptoms defining disturbance of attention, hyperactivity, and impulsivity, but also of emotional dysregulation, are frequent and similar to items in ASRS-v1.1, the items of PQ-16 center more on symptoms of delusions and boundary disturbances.

The internal consistency reflecting the homogeneity of the scales was for nearly all instruments moderate to high, with only ASRS-6 having a small Cronbach’s α. In comparison, ASRS-18 showed a high internal consistency. These results are similar to of Buchli-Kammermann et al. (30). SPro had the highest internal consistency of all instruments, exceeding Kammermann et al.’s (27) estimate.

All correlations between the total scores of the instruments were statistically significant. As expected, the correlations of scales defining similar constructs were all in all stronger than those defining different constructs. However, these correlations were only slightly stronger, raising the question whether these scales are not to unspecific to distinguish between ADHD and psychosis risk symptoms. The correlation of SPro *psychosis risk* and ASRS-18 were strong, although these scales define two different construct. The correlations between ASRS-6 and ASRS-18, between SPro *psychosis risk* and SPro were strong as well because they share some identical items. The convergent and the divergent validity are at the end not satisfying.

The balance of the SENS, SPEC, PPV, and NPV in the ROC analyses was modest. The purpose of a screening instrument is to identify as many individuals as possible with a specific set of symptoms, i.e., the SENS of a screening instrument should preferably be high compared to the SPEC. Only the SPro and its subscale *psychosis risk* meet this requirement. The SENS of the other instruments was mostly higher than the SPEC, indicating they were better able to correctly identify subjects presenting with neither psychotic nor ADHD symptoms. Nonetheless, the AUC values discriminating between positive and negative cases were statistically significant in all comparisons, centering around 0.7, with a value of 0.5 indicating no difference, and 1.0 a perfect separation of two specific groups.

Compared to ASRS-6, ASRS-18 showed the highest OR to achieve its own COV and that of an instrument measuring psychosis risk symptoms. Consequently, the symptomatic overlap between ASRS-18 and the SPro subscale *psychosis risk* seems the most important, indicating that ASRS-6 is better able to detect individuals with ADHD symptoms and to be preferred. As to psychotic symptoms, SPro had the smallest OR and is to be preferred for identifying subjects at risk of psychosis. The OR results for instruments defining similar constructs were as expected: the probability for an instrument to achieve the COV of another questionnaire was higher for scales determining similar rather than different aspects.

The analyses of the four diagnostic groups showed patients with ADHD as well as psychotic symptoms had a higher symptomatic overlap than the others, both in the sum and sub scores. Subjects with either ADHD or psychotic symptoms were not better identified with the specific questionnaires.

Although the instruments used were all found reliable and valid in previous studies analyzing their psychometric properties (26–29), they are shown here not to be reliable enough to separate symptoms of ADHD from those of psychosis risk. The purpose of a screening instrument is to filter out patients with suspicious symptoms and to initiate timely and appropriate diagnostic procedures and interventions. Unfortunately, the present results fail to clarify which instruments are better able to identify patients with ADHD or psychosis risk symptoms. The reason might lie in the difficulty to distinguish between the—to some extent nonspecific—symptoms of these two conditions and in the complexity of symptoms of both ADHD and psychosis risk (33). Consistent with other studies (2, 3, 7, 17), this study confirms the overlap between ADHD and psychosis risk symptoms possibly reflecting similar characteristics between ADHD and psychosis risk [environmental risk factors and neurobiological substrates (3)]. The aim of a systematic screening process is to allow the identification of risk patients, here also to prevent or delay transition to psychoses and so improve prognosis and save treatment costs (34). The evident symptomatic overlap makes it difficult to refer patients to a special consultation unit for a specific diagnostic procedure allowing for suspected ADHD or psychoses either to be confirmed or dismissed. This underlines the need for sensitive screening instruments in this specific field.

### Limitations

There are several limitations to these findings. The use of self-rating scales only could be problematic for both patients experiencing symptoms of psychosis risk (35) and individuals with ADHD symptoms (36). Additional observer-rating scales could help to better understand the symptomatic overlap between ADHD and psychosis risk and enable the comparison of results from different sources of information. Another possible weakness of the study is the small number of patients presenting with an indication of ADHD only. This may lead to an inflation of the present results, giving individuals with only psychosis risk symptoms or with ADHD and psychosis risk symptoms more weight in the statistical analyses.

It will be important to replicate the present study by extending the method of assessing ADHD and psychosis risk symptoms in a randomized, controlled trial. The assessment and analysis of other psychiatric and comorbid disorders of the participants is to consider and is highly recommendable, in order to make more accurate statements. Finally, it is questionable whether the COV proposed by Ramos-Quiroga et al. (31) and adopted in this study is also valid in a German-speaking sample.

## Conclusion

To the best of our knowledge, the present study is the first to analyze the ability of ASRS-v1.1, SPro, and PQ-16 in a heterogeneous adult population seeking psychiatric treatment. The ability of the three different instruments to distinguish between patients at risk of ADHD or psychosis is found to be modest. The symptomatic overlap between these two conditions evidently makes it difficult to identify either disorder correctly and to give definitive advice on initiating specific diagnostic procedures. There is a need for reliable and sensitive screening instruments able to clearly separate symptoms of ADHD from those of psychosis to prevent further suffering and impairments in major life activities for patients at risk of these conditions and to reduce treatment costs.

## Ethics Statement

This study was carried out in accordance with the recommendations of Ethics Committee of Basel (Ethikkommission Nordwest- und Zentralschweiz) with written informed consent from all subjects. All subjects gave written informed consent in accordance with the Declaration of Helsiki. The protocol was approved by the Ethics Committee of Basel (Ethikkommission Nordwest- und Zentralschweiz).

## Author Contributions

All authors contributed extensively to the work presented in this paper.

## Conflict of Interest Statement

The authors declare that the research was conducted in the absence of any commercial or financial relationships that could be construed as a potential conflict of interest.
